# Loop Mediated Isothermal Amplification: Principles and Applications in Plant Virology

**DOI:** 10.3390/plants9040461

**Published:** 2020-04-06

**Authors:** Stefano Panno, Slavica Matić, Antonio Tiberini, Andrea Giovanni Caruso, Patrizia Bella, Livio Torta, Raffaele Stassi, Salvatore Davino

**Affiliations:** 1 Department of Agricultural, Food and Forest Sciences, University of Palermo, 90128 Palermo, Italy; andreagiovanni.caruso@unipa.it (A.G.C.); patrizia.bella@unipa.it (P.B.); livio.torta@unipa.it (L.T.); stassiraf@gmail.com (R.S.); 2 Department of Agricultural, Forestry and Food Sciences, University of Turin, 10095 Turin, Italy; slavica.matic@unito.it; 3 Council for Agricultural Research and Economics, Research Center for Plant Protection and Certification, 00156 Rome, Italy; antonio.tiberini@crea.gov.it; 4 Institute for Sustainable Plant Protection, National Research Council (IPSP-CNR), 10135 Turin, Italy

**Keywords:** loop-mediated isothermal amplification, LAMP, *Bst* DNA polymerase, primers, real-time monitoring, virus, viroids, plant virology

## Abstract

In the last decades, the evolution of molecular diagnosis methods has generated different advanced tools, like loop-mediated isothermal amplification (LAMP). Currently, it is a well-established technique, applied in different fields, such as the medicine, agriculture, and food industries, owing to its simplicity, specificity, rapidity, and low-cost efforts. LAMP is a nucleic acid amplification under isothermal conditions, which is highly compatible with point-of-care (POC) analysis and has the potential to improve the diagnosis in plant protection. The great advantages of LAMP have led to several upgrades in order to implement the technique. In this review, the authors provide an overview reporting in detail the different LAMP steps, focusing on designing and main characteristics of the primer set, different methods of result visualization, evolution and different application fields, reporting in detail LAMP application in plant virology, and the main advantages of the use of this technique.

## 1. Introduction

### 1.1. Advances in Plant Virus Diagnostics

The year 1977 represents the watershed for diagnosis in plant virus diseases (or virus diagnostics). In fact, in this year [1], the first enzyme-linked immunosorbent assay (ELISA) method was published for the detection of plum pox virus (PPV) and arabis mosaic virus (ArMV). This technique projected diagnosis in plant virology into a new era. Before the ELISA method, the diagnosis of plant virus diseases was exclusively carried out by specialists with many years of experience in description and collection of virus symptoms in different hosts. Furthermore, the few known techniques were based on complex, expensive, and time-consuming techniques, such as bio-tests on indicator plants. Since its introduction, ELISA has revolutionized virus diagnosis, thanks to short detection time, especially if related to indexing on indicator plants. In a few years, the ELISA method has become the major tool for virus diagnosis in different application fields, such as plant disease diagnosis, breeding, quarantine, and certification program [2].

Another important step forward in plant virology diagnosis was the introduction of the nucleic acid-based techniques, especially polymerase chain reaction (PCR) [3]. This technique allows the detection of pathogens such as viroids, different phytoplasmas, or viruses, for which antibodies are not available. Moreover, PCR-based methods give the possibility to perform single or multiplex assays [4,5,6,7]. To date, from the first paper that described PCR methods for virus detection in 1990 [8], 574,939 papers have been published in PubMed [9]. As well as the ELISA test, the PCR technique is widely used in diagnostic laboratories, although it has numerous limitations, such as (i) expensive analysis equipment; (ii) requirement of highly specialized personnel; (iii) different rooms for samples preparation; (iv) post-PCR contamination; and (v) cost per single analysis approximately double than an ELISA test. All these problems can be bypassed for routine diagnosis through the use nucleic acid hybridization-based methods [10], although this technology has not been very successful owing to limited availability of specific probes. A milestone for diagnosis in plant virology was the real-time PCR-based method, initially developed for gene expression analysis in genetic studies, and subsequently for diagnostic applications [11,12,13,14]. However, the high cost of the real-time thermocycler, as well as the requirement for laboratories with separate rooms and extremely skilled personnel, represented the main limiting factors that did not allow this technique to become a routine technique. In this context, it becomes necessary to identify other techniques that relate to the ease of use of ELISA, and the accuracy of PCR.

A significant step forward in this direction has been made with the development of isothermal amplification techniques, of which loop mediated isothermal amplification (LAMP) [15] is certainly the one that is having the greatest success today.

### 1.2. Loop Mediated Isothermal Amplification (LAMP)

LAMP is a nucleic acid amplification method initially designed and developed by Notomi and coworkers [15] to amplify a specific DNA region of hepatitis B virus (HBV) under isothermal conditions. The procedure enabled a fast, sensitive, and specific detection of a selected target opening new possibilities in the diagnostic field. Since this first report, the application of LAMP has been increasingly used and adopted as an alternative method to those based on PCR. LAMP, in fact, is continuously being implemented in the medicine, agriculture, and food industries, with approaches that include the screening of viral and bacterial strain mutations, analysis of fungicide resistant mutations, analysis of micro RNAs, herbal medicine identification, plant pathogen vectors identification, single nucleotide polymorphisms analysis, and detection of genetically modified organisms [16,17,18,19,20]. The reasons for the development of the LAMP methodology were founded on the attempt to overcome some drawbacks of the conventional PCR, a method that requires the acquisition of a high-cost equipment known as thermal cycler. The necessity of a high precision in heating/cooling ramps and temperatures produces occasional losses of the appropriate specificity for the identification of the selected targets [15]. In addition, the polymerase enzyme is quite sensitive to inhibitors usually present in nucleic acid extracts, especially isolated from plant matrices [21]. Conversely, the isothermal amplification by LAMP does not require any specific equipment, while it provides high specificity owing to the use of four to six primers that recognize between six and eight independent regions, all of them addressing a specific target region. In addition, the robustness of the enzyme used in the LAMP methodology reduces the inhibitors problems [15,22,23,24]. The benefit of LAMP to be easily adapted for point-of-care analysis makes the technique a valid method for surveys or quarantine programs where rapid, reliable, and specific analysis is required.

## 2. Principles of LAMP

The LAMP technique is based on auto cycling and high DNA strand displacement activity mediated by *Bst* polymerase from *Geobacillus stearothermophilus*, under isothermal conditions. The reaction consists of two steps: an initial step and a combination of a cycling amplification step with an elongation/recycling step [22]. The isothermal amplification is carried out at 60–65 °C, the optimum temperature for *Bst* polymerase activity [25]. In the pioneering study of Notomi and coworkers [15], the reaction of LAMP employed a set of four primers that were able to recognize six different sequences within the target HBV viral DNA. The four primers contained two inner primers (forward inner primer (FIP) and backward inner primer (BIP)) and two outer primers (forward outer primer (F3) and backward outer primer (B3)). Inner primers consisted of two different sequences that recognized a sense and an antisense sequence of the target DNA, while the outer primers recognized only one external sequence of the target DNA [15].

### 2.1. General Considerations

#### 2.1.1. Initial Step

**Figure 1:**
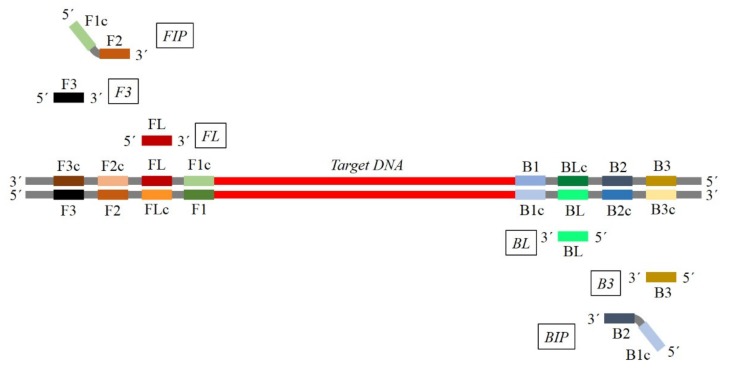
Typical map for loop mediated isothermal amplification (LAMP) primer set positioning and corresponding sequence homology. For each primer, the same color represents the corresponding sequence in the target. The primer sequence corresponds to the reverse complementary sequence of the target region. (FIP: forward inner primer; F3: forward outer primer; FL: loop primer F-Loop; BL: loop primer B-Loop; B3: backward outer primer; BIP: backward inner primer).

The initial step is performed at 65 °C, at this temperature, primers are able to anneal to the specific sequence [15,23]. The forward inner primer hybridizes with the original reverse target sequence and the synthesis of the new forward strand starts from the 3′ end flanked by the forward inner primer. Then, the forward outer primer hybridizes again with the same original reverse target sequence and the synthesis of this new forward strand continues until the enzyme finds the 5′ end of the first strand created with the use of the inner primer (Figure 1). Subsequently, owing to the properties of the enzyme used in LAMP, the strand displacement of the first forward strand created by the use of the forward inner primer occurs. This separated strand creates a self-hybridizing loop at one end owing to the complementarity of the reverse sequence from the inner primer to the target sequence. It also serves as a template for the reverse inner primer and the reverse outer primer, which, in a similar way, will produce the strand displacement of the forward strand, thus creating a dumbbell-like DNA structure [15,22].

#### 2.1.2. Cycling Amplification and Elongation Step

During this phase, the forward inner primer hybridizes to the loop of the strand formed during an initial step and facilitates the strand displacement to generate a new strand with an inverted copy of the target sequence in the stem region and a loop at the opposite side. Subsequently, self-primed strand displacement DNA synthesis results in two products, one complementary strand and another strand with double elongated stem as long as the original and the loop on the opposite site. Both strands are then used as templates for the reverse-primed strand displacement synthesis in the subsequent elongation and recycling steps. This allows a threefold amplification of the target sequence at each half of the cycle [15,22]. Owing to the high displacement activity of the *Bst* DNA polymerase, a huge amount of DNA with a high molecular weight is rapidly generated [26,27,28]. This allows target DNA amplification until 10^9^ copies in less than one hour. Finally, the stem-loop DNAs of the different length and cauliflower-like structures having multiple loops are generated [29].

#### 2.1.3. LAMP Acceleration

In order to speed up the reaction two additional primers, named loop primers (loop forward (LF), loop backward (LB)) could be included [24]. These primers hybridize with the stem-loops, with an exception of the loops hybridized by the inner primers and prime strand displacement DNA synthesis [24]. Currently, six primers are widely used in many diagnostic protocols to allow better specificity and sensitivity [30,31,32].

Another implementation of this methodology was based on the ability of the *Bst* polymerase to tolerate different types of inhibitors [33]. Thus, several protocols in plant pathology were developed, where crude plant saps were used as template. This implementation accelerated the time for the diagnosis, avoiding any previous step for samples preparation and DNA or RNA purification [26,34,35,36,37,38,39]. In that way, diagnosis time was speeded up for 2–3 h, which was necessary to complete the extraction of DNA/RNA of target organism. At the same time, the cost of the LAMP reaction is importantly reduced without the need for expensive nucleic acid extraction kits [36,40]. In fact, a LAMP test costs about $3, making this test very inexpensive and widely usable, especially when compared with the costs of a conventional PCR end point, which requires a cost of at least $12, as it is necessary to perform nucleic acid extraction, PCR assay, and electrophoresis.

## 3. General and Specific Considerations for Primers Design

In all the nucleic acid amplification-based methods, primers and/or probes design is one of the most critical factors affecting the success and quality of the results [41]. The LAMP primers design requires the selection of eight different regions of the target nucleic acid sequence.

Significant attention must be paid in order to avoid dimers formation among primers, especially for FIP and BIP primers, which are generally about 40 nucleotides long. FIP consists of the F2 sequence (at its 3′ end) that is complementary to the F2c region, and the same sequence as F1c region at its 5′ end. BIP consists of the B2 sequence (at its 3′ end) that is complementary to the B2c region, and the same sequence as B1c region at its 5′ end. LF primer is designed using the complementary strand corresponding to the region between F1 and F2, while LB primer is designed using the complementary strand corresponding to the region between B1 and B2 [15] (Figure 1).

### Key Factors for Primer Design

Several key factors have to be considered for designing primers, such as primer length, % Guanine and Cytosine (GC) content (optimal GC content range between 40% and 60%), no-specific binding, stability at 3’ and 5’ end of each primer, primer secondary structures, and melting temperature (Tm). In addition, as multiple primers anneal in different target regions at the same time, it is necessary consider the distance between primers, primer secondary structures (self-dimers and cross dimers), and Tm balance.

## 4. LAMP Primer Design Software

Owing to the complexity of primer design, several softwares have been developed and could be retrieved online, both free or licensed. In the literature, the most common used is Primer Explorer V4 (PE4), a free primer design software specific for the LAMP technique, supplied by Eiken Chemical Co. [42].

Other softwares, aimed to implement and improve different features, have been developed, but most of them are expensive and covered by license. Among them, LAMP Designer (Optigene, Horsham, England) and LAVA (LAMP Assay Versatile Analysis) [43] allow to avoid cross homologies while designing the primers, and permit primer design by multiple sequences alignment.

## 5. Sample Preparation Methods for Viruses and Viroids Detection

Starting from the assumption that the total DNA/RNA extraction for viruses and viroids detection it is carried out through the use of commercial kits, in order to reduce costs per single reaction and speed up the analysis, different sample preparation methods could be performed. Below are reported some sample preparation methods that simultaneously allow to reduce costs and performing times. Immunocapture in microtubes—in this procedure, typical 0.2 mL tubes are incubated at 37 °C for 1 h with a specific amount of monoclonal or polyclonal antibody for the specific pathogen detection diluted in a coating buffer for Double antibody sandwich-enzyme-linked immunosorbent assay (DAS-ELISA). Subsequently, after three washing steps, 100 µL of sap extract (obtained grinding the petioles or other plant portion in a typical extraction buffer for DAS-ELISA) is added. After 1 h of incubation at room temperature, the tubes are washed with washing buffer, dried, and prepared for the LAMP assay [12,44]. Direct crude extract—a slice of 0.4 mm of the plant portion is directly placed in a 1.5 mL tube containing 0.5 mL of glycine buffer (EDTA 1 mM, NaCl 0.05 M, glycine 0.1 M), vortexed for 30 s, and heated at 95 °C for 10 min. Three microliters of this mixture are directly used for the LAMP assay [12]. Leaf-disk crude extract—five or ten fresh-cut petioles are impressed in a one cm^2^ of hybridisation membrane, dried at room temperature for 5 min, and placed in a 1.5 mL tube containing 0.5 mL of glycine buffer. Subsequently, tubes are vortexed for 30 s and heated at 95 °C for 10 min. Then, 3 μL of obtained mixture is used for the LAMP assay [12,45]. Spotting on membrane disks—samples are prepared in extraction buffer and, subsequently, 10 µl of this extract is spotted onto 5 mm diameter nylon membrane disks; dried at room temperature for 5 min; placed in a 1.5 mL tube containing 100 μL of glycine buffer; and heated at 95 °C for 10 min, vortexed, and placed on ice. Aliquots of 2 to 10 μL are used for the reaction [46].

## 6. Reverse Transcription (RT)-LAMP

From the year 2000, when Notomi developed the LAMP method, different companies developed LAMP-based kits, including their own polymerase and buffer, allowing to personalize, according to experimental conditions, only the primers set concentration. Moreover, the LAMP technique was implemented by an additional step including a reverse transcription step (RT-LAMP), in a one-step protocol where the specific enzyme, usually the avian myeloblastosis virus (AMV) reverse transcriptase, is included. The reverse transcriptase enzymes were modified to perform the reaction at a higher temperature range required for the isothermal amplification (60–65 °C) [47]. One of the first applications for a one-step RT-LAMP in plant pathology was reported in 2005 by Nie [48] for the detection of potato virus Y (PVY); from that time, several one-step assays have been reported in the literature (see Table 1).

## 7. Visualization Methods of Amplification Products

A large amount of target DNA, as well as a large amount of by-product, is produced during the continuous amplification under isothermal condition of a LAMP reaction.

There are two groups of visualization result methods, one based on reading results at the end of the amplification process (end point-based methods), and a second one that is based on reading results during the process (real-time-based methods). As the method is characterized by a high sensitivity and allows a simple visual reading of the reaction results (naked-eye), almost all of the visualization methods are based on end point methods, including agarose gel electrophoresis, observation of color change of the reaction mixture, and using ion indicators, which cause the turbidity change of the mixture [146], or fluorescent molecules, such as ethidium bromide (EtBr) [24] or SYBR Green I [147].

Regarding the new methods of results visualization in real time, today, it is possible to measure the turbidity [148,149] or the fluorescence of particular substances, for example, SYBR Green I [150], during the amplification process, without waiting until the end of the process (real-time-based methods).

### 7.1. End Point-Based Methods

Different LAMP end point methods are currently available, among which the most commonly used are listed below. Furthermore, many commercial kits based on the LAMP method were developed and adopted for routine identification, surveillance, and diagnosis of human, animal, and plant pathogens worldwide [30,151,152].

#### 7.1.1. Agarose Gel Electrophoresis

**Figure 2:**
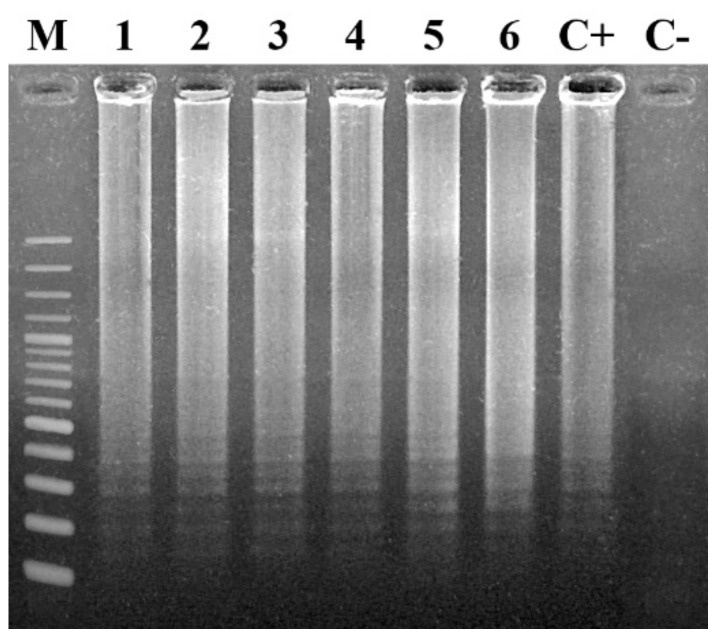
Typical electrophoretic analysis on a 2% agarose gel followed by ethidium bromide (EtBr) staining of LAMP amplified products, using a specific primer set. The LAMP method forms amplified products of various sizes (“ladder pattern”), consisting of alternately inverted repeats of the target sequence on the same strand. Lane M: 1 kb ladder (Nippon Genetics); lanes 2–6: positive LAMP reaction samples; lane C+: positive control; lane C−: negative control.

Generally, 5 μL of LAMP products is visualized under UV light after electrophoresis on 2%–3% agarose gel in Tris-borate buffer. Gel staining can be carried out using different intercalating dyes, such as SYBR Green I [153,154], EtBr [155], Picogreen [156], or propidium iodide [157]. Regardless of the intercalating dyes used for the visualization of LAMP products, the result is represented by a ladder-like pattern on the gel, caused by the formation of stem-loop DNAs of varying stem length and cauliflower-like structures with multiple loops formed by sequentially inverted repeats of the target sequence [156] (Figure 2). Smeared DNA between bands and at the well was shifted to bands of <10 kb when the products were analyzed by alkaline agarose gel electrophoresis [15].

LAMP is an extremely sensitive reaction and could lead to incorrect results upon contamination of even a small quantity of DNA amplification products. In order to avoid the very high risk of contamination, caused by handling LAMP-amplified product, reagents and reaction mixtures should be prepared in a different place where the reaction product tubes are opened to perform electrophoresis [23].

It is also possible to determine the amplification specificity by restriction digest of LAMP products.

In order to simplify the application of the LAMP technique in the field, the result of amplification can also be visualized by naked-eye inspection, evaluating the fluorescence or turbidity of the sample.

#### 7.1.2. Fluorescent Dyes

In order to facilitate the field application of the LAMP assay, the monitoring of amplification could be carried out through naked-eye inspection [21]. The inspection for amplification can be performed through observation of a color change following addition in the reaction mixture of a specific fluorescent dye, such as EtBr [158] SYBR Green I [149], Picogreen [156], and propidium iodide [157]. Fluorescent dyes are added directly into the reaction mixture and the amplification results can be visualized without opening the reaction tubes, decreasing the risk of contamination. The fluorescent molecule linked to DNA does not affect the activity of other elements in the reaction mixture, such as enzymes.

**Figure 3:**
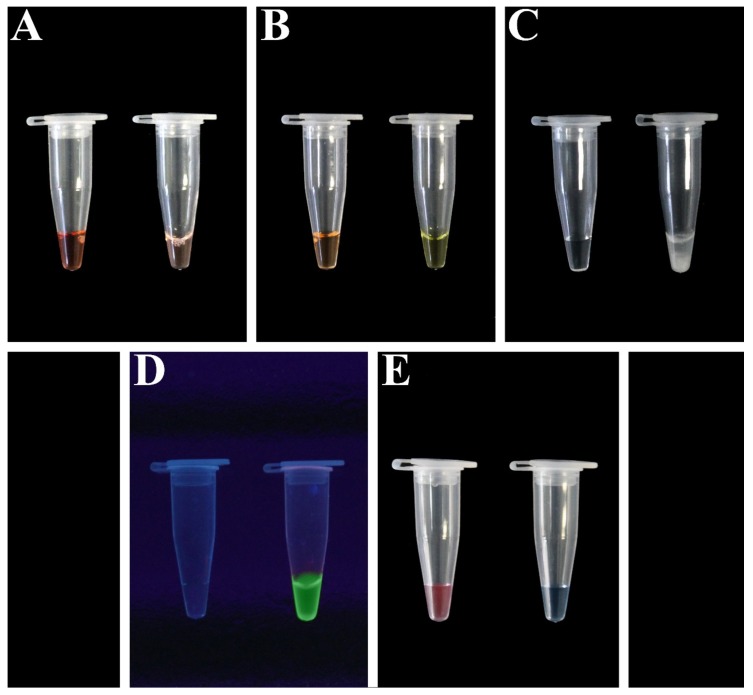
Different naked-eye results inspection. (**A**) Naked-eye observation under natural light of the reaction tube after addition of ethidium bromide in the reaction mixture for positive result discrimination. In the case of a negative result, a wine coloration is shown (left), while in a positive sample, the reaction mixture shows a salmon pink coloration (right). (**B**) Naked-eye observation under natural light of the reaction tube with Picogreen fluorescent dye in LAMP assay amplification. In the case of a negative result, the original orange color is retained (left), whereas in the case of a positive amplification, the original color of the dye changes permanently to yellow (right). (**C**) Result discrimination by ion indicator: (left) negative control that did not contain template DNA, showing clear mixture; (right) increases in turbidity observed on positive sample. (**D**) Detection of target DNA in LAMP reaction under UV light, using calcein as fluorescent metal indicator. (Left) negative control; (right) emitted fluorescence by positive sample owing to the interaction of calcein with residual magnesium ion. (**E**) Result discrimination by HNB (hydroxy naphthol blue): the color change of the mixture is from violet on the negative reaction (left) to sky blue on the positive reaction (right).

The addition of EtBr in the reaction mixture (1 mM in a final volume reaction of 25 µL) [159] causes a salmon pink coloration of the mixture in the case of a positive reaction, while a wine coloration is observed in a negative reaction (Figure 3A). It is necessary to wear gloves while handling, because ethidium bromide is a known mutagen and must be handled as a hazardous chemical [23].

SYBR Green I or Picogreen dye (the final dilution of 1:1.000) change the mixture color from the original orange to permanent yellow in the case of positive amplification (Figure 3B). The results can be visualized under natural light without any equipment as well as under UV light. In absence of amplification, the original orange color of the dye is retained [149,156]. The main advantages of SYBR Green I dye and Picogreen dye are as follows: greater detection efficiency of nucleic acids, higher detection limit than other most used compounds, less dangerous compared with ethidium bromide, and lower management costs compared with other compounds. Further, in this case, the fluorescent molecule does not affect the activity of other compounds in any way.

Similar to SYBR Green I and Picogreen dyes, the identification of a positive sample using propidium iodide does not require any special processing or electrophoresis, just a naked-eye visualization of the color change of the reaction mixture at ambient light. Further, in this case, the result visualization can be further improved using UV light. Usually, 1 µL of the diluted dye (1:10 dilution of 10 mg/mL stock solution) is added to 25 µL of the reaction mixture to develop the color reaction [157]. The color changes from a deep red-orange in the negative reaction to a light (almost clear) pink in the positive reaction. Compared with SYBR Green I, propidium iodide does not require freezing for storage, and it is less expensive [157].

#### 7.1.3. Ion Indicators

In some cases, the use of expensive equipment decreases the versatility of LAMP and greatly limits the wide use of this procedure, especially in developing countries.

Generally, during the DNA polymerization process, which takes 30–60 min, a huge amount of magnesium pyrophosphate is produced as by-product generated from a combination of pyrophosphate ion released from the substrate (deoxyribonucleotide triphosphates (dNTPs)) and magnesium ion in the reaction mixture [146]. The production of magnesium pyrophosphate precipitates produced as a result of combination with magnesium ion in the reaction solution is schematically represented by the following reaction:

$${\left(DNA\right)}_{n-1}+dNTP\to \;{\left(DNA\right)}_{n}+P_{2}O_{7}^{4-}\;P_{2}O_{7}^{4-}+2Mg^{2+}\to \;Mg_{2}P_{2}O_{7\;}$$

Consequently, the metal ion concentration will significantly decrease, and the LAMP reaction can be monitored by observing the formation of white precipitates (Figure 3C) [146]. However, the visual detection of the results is particularly difficult and linked to the operator’s experience.

It is possible to centrifuge the tube with amplified DNA at 6000 rpm for several seconds, to accumulate precipitates at the bottom of the tubes, in order to simplify the result visualization by the naked-eye. Therefore, it is necessary to detect the change in metallic ion concentration during the LAMP reaction, obtaining a clearer signal.

**Figure 4:**
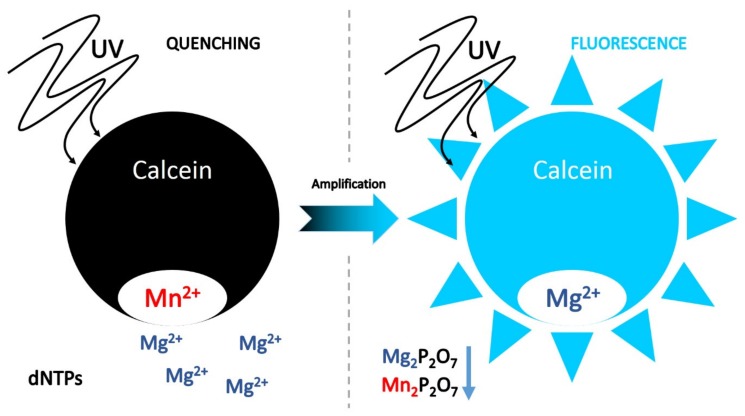
Detection using calcein as a fluorescent metal indicator. In the DNA amplification process, pyrophosphate ions are produced as a by-product from the reaction substrate deoxyribonucleotide triphosphates (dNTPs). In the presence of target DNA, during the LAMP reaction, newly generated pyrophosphate ion (P_2_O_7_^4−^) deprives calcein of manganous ion, which combines with residual magnesium ion (Mg^2+^), producing greater fluorescence.

As described by Tomita and co-workers [23], calcein combined with manganous ion can be used as a fluorescent metal indicator, in order to quench the reaction for the detection system of the LAMP method (Figure 4). Calcein is a metal indicator that yields strong fluorescence by forming complexes with divalent metallic ions, such as magnesium and calcium. In presence of target DNA, during the LAMP reaction, newly generated pyrophosphate ion deprives calcein of manganous ion, which combines with residual magnesium ion, producing greater fluorescence (Figure 3D).

Another metal ion indicator, namely hydroxy naphthol blue (HNB), could be used in a colorimetric assay for the detection of the LAMP reaction. HNB is a metal indicator for calcium and a colorimetric reagent for alkaline earth metal ions. This detection system allows a great discrimination of a positive reaction, indicated by a color change (from violet to sky blue, Figure 3E) [160]. In detail, 120 μM HNB is pre-added on the reaction mixture solution and does not inhibit the amplification efficiency or interfere with DNA synthesis. Similar to other metal ion indicators, it is not necessary to open the reaction tube to determine whether the reaction was positive or negative, reducing the risk of cross-contamination. The LAMP reaction with HNB can be carried out in both PCR tubes or in a 96-well microplate. The results could be easily judged both by naked-eye inspection or by absorbance measurement (650 nm) with a microplate reader. The color change of positive/negative samples is stable after two weeks of exposure to ambient light, while higher absorbance values in the positive reactions are caused by the precipitation of the by-product magnesium pyrophosphate in the optical path.

The results obtained have shown a detection sensitivity equivalent to SYBR Green I [160]. The detection systems just described, on the basis of visual discrimination of results, could be extremely helpful for point-of-care testing, clinical diagnosis, environmental measurement, agriculture and so on. Compared with other nucleic acid amplification techniques, the main advantages are the absence of expensive specialized equipment, ease of operation, good sensitivity and speed, low contamination risk, and helpfulness for both DNA and RNA detection.

### 7.2. Real-Time-Based Methods

The continuous improvement of the LAMP technique has contributed to developing new real-time-based methods (real-time LAMP). Currently, the most common real-time LAMP methods are based on the turbidity measurement of the reaction mixture, owing to the formation of precipitates, as previously described, or on fluorescence measurement emitted by intercalating substances introduced into the reaction mixture, such as ethidium bromide or SYBR Green I.

DNA template it is detected in the LAMP reaction, and can also be quantified, optimizing the reaction conditions and primers. For this reason, Mori and coworkers [148] developed a turbidity measurement apparatus (real-time turbidimeter) that allows to measure in real time the turbidity of the reaction during isothermal amplification. Thus, this method permits to maintain the optimum temperature (60–65 °C), to continuously and simultaneously measure the turbidity of multiple samples, using typical 0.2 mL PCR tubes. In the year 2008, an integrated isothermal device was developed for both amplification and detection of targeted HBV DNA from clinical serum samples by kinetic analysis of LAMP reactions [161].

This method has two important advantages: there is no need to check the amplification product at the end of the reaction by naked-eye, and the ease to read the turbidity of the amplification product by real-time turbidimeter. This method permits to measure the turbidity in order to monitor the DNA amplification; the relationship between turbidity and the time curve represents the reaction curve of the LAMP amplification.

**Figure 5:**
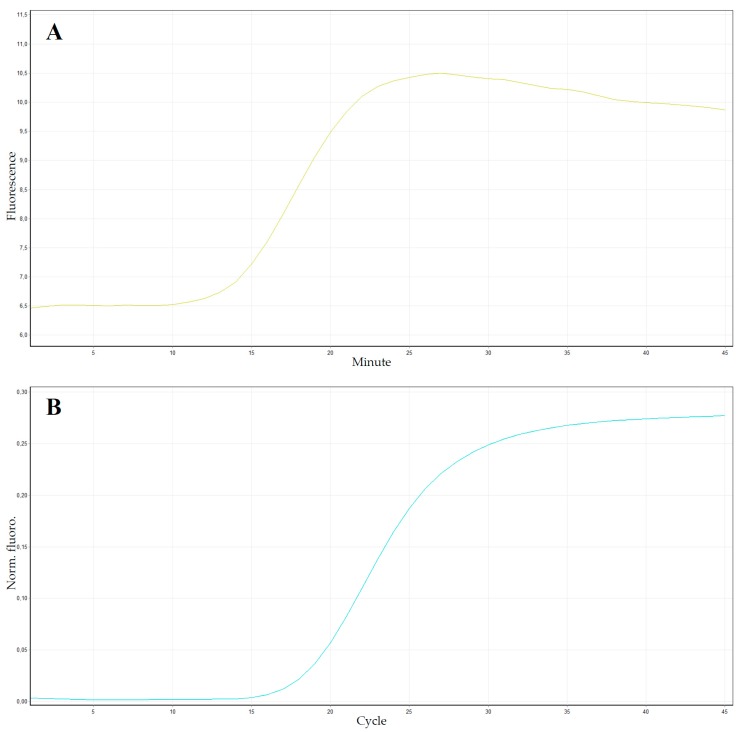
Result comparison between real-time LAMP and conventional real-time polymerase chain reaction (PCR). Panel (**A**) shows the LAMP amplification curve, which looks like a “hat”, while panel (**B**) shows the conventional sigmoidal real-time PCR amplification curve.

It is possible to implement another real-time approach by adding a fluorescent dye, such as SYBR Green I, into the reaction mixture. As reported by Maeda and coworkers [150], SYBR Green I can be used for real-time monitoring of the LAMP reaction, as a source of fluorescence. This method shows almost identical results to the conventional real-time PCR (Figure 5B) with an advantage of rapidity and simplicity. The quantitative detection can be carried out using a typical real-time PCR thermocycler. The increase in fluorescence caused by dsDNA-binding SYBR Green I is monitored every 30–60 sec for 40–60 min under isothermal condition (60–65 °C). In routine experiments, the real-time LAMP assay generates results in <30 min for most samples.

Another fluorescent molecule that could be used for real-time monitoring is ethidium bromide. For the first time, EtBr was used by Nagamine and coworkers [162] in a LAMP assay, adding 0.25 µg/mL of EtBr into the total 25 μL reaction mixture, measuring the fluorescence intensity using the ROX (6-Carboxyl-X-Rhodamine) fluorescence channel [24]. An increase in fluorescence is directly correlated with the DNA amplification. In some cases, EtBr gave a better detection signal than SYBR Green I [163]. Contrary to classical real-time PCR, when EtBr or SYBR Green I are used in a real-time LAMP assay, the amplification curves are not sigmoidal, but rather look like a hat (Figure 5A) [163]. In fact, during DNA amplification, the fluorescence increase is followed by a simultaneous increase of magnesium pyrophosphate precipitates, causing a turbidity increase, which masks the fluorescence. This effect might be more relevant at the end of the process, with a fluorescence diminution detection.

## 8. LAMP Application in Plant Virology

Many plants, such as vegetable and herbaceous crops, fruit trees, and weeds, are affected by viral and viroidal diseases, which cause considerable economical losses worldwide. Therefore, it is extremely important to develop rapid and accurate detection methods, in order to accelerate the diagnosis process and apply appropriate intervention measures, especially in agro-ecological contexts, such as intensive cultivations. During the last years, detection of these agents became challenging. The wide use of LAMP technology has significantly facilitated this task. Currently, LAMP has an important influence in diagnosis, with relevance in plant virology. The first LAMP protocols in phytopathology were reported in 2003 by Fukuta and coworkers for the detection of two plant viruses on horticultural crops, tomato yellow leaf curl virus (TYLCV; Circ-ssDNA+/−) [139] and japanese yam mosaic virus (JYMV; ssRNA+) [84]. Subsequently, different protocols were developed for different plant viruses and viroids including economical and agronomical important agents. In detail, as reported in Table 1, from 2003 to today, many authors have developed different LAMP protocols for the detection of 100 viruses, belonging to 23 different families and 47 genera. Furthermore, RT-LAMP protocols were developed for eight viroid species, belonging to two families and four genera (Table 1).

Recently, the development of LAMP portable devices has allowed a ‘real-time’ detection pathogen directly on-field [39,164], facilitating their diagnosis during the routine surveys or in sanitary selection or eradication programs.

## 9. LAMP: Evolution and Recent Applications

In order to overcome the limits of classical diagnostic methods (i.e., culture methodology and microscopy), various molecular diagnosis methods have been developed, such as PCR, real-time PCR [12], molecular hybridization [165], flow-through hybridization [10], ligase chain reaction (LCR), nucleic acid sequence-based amplification (NASBA) [166], SDA [167], rolling circle amplification (RCA) [168], single primer isothermal amplification (SPIA), and circular helicase dependent amplification (cHDA) [169]. Many of them have been marketed as commercial kits and used in routine diagnostics. The main shortcomings include expensive equipment and reagents required, specialized personnel, and equipped laboratories; consequently, these techniques cannot be used in developing countries.

In recent years, LAMP technology has been applied in many research and diagnosis fields. Initially, research and development focused on LAMP application in a clinical setting, in order to improve existing diagnostic tests. More recently, it has been used in basic research to simplify LAMP assays as much as possible [154] and for testing the distinct features of the technique. As mentioned above, especially in recent years, LAMP has allowed the development of various phytopathological diagnosis protocols for the early detection of many diseases caused by viruses and viroids (see Table 1).

It is necessary to improve and integrate LAMP technology into simple genetic tests in order to be used as point-of-care diagnostics [170]. In fact, the LAMP technique is a fundamental method for the identification and diagnosis of different pathogens, and is extremely useful for control and quarantine inspection of important human, animal, and plant diseases. In addition, LAMP is used to detect genetically modified products, as well as for cancer and embryo sex identification [47].

Over the past 20 years, since the LAMP technique was developed by Notomi and coworkers in 2000 [15], it has been significantly improved, adapting it to different contexts, and also leading to the development of new diagnostic tests, as well as for new research and development strategies. Once the effectiveness and the applicability in various diagnostic fields was demonstrated, the researchers turned their attention to LAMP application in poorly equipped laboratories with low resource settings, to offer developing countries a rapid diagnostic method with the following characteristics: reliability, sensitivity, simplicity, specificity, and easy availability of simple instruments [171].

A great LAMP characteristic is that it can be carried out using a common block heater or water bath, but these kinds of tools require electricity. In many contexts, this can be a critical barrier to timely diagnosis. For this reason, LaBarre and coworkers (2011) [172], in order to simplify the heating method for the LAMP reaction, have designed an electricity-free heater based on exothermic chemical reactions and engineered phase change materials that can be used for any kind of isothermal nucleic acid amplification assays, such as LAMP. Major improvements of this technique are based not only on its application in unscientific or developing contexts, but also on the research and development background. Through recent years, different “versions” of LAMP have been developed.

### 9.1. Multiplex LAMP (mLAMP)

In 2007, Iseki and coworkers [173] developed a mLAMP technique that allows the simultaneous detection of two pathogens; it is based on combining two sets of LAMP primers (totaling eight primers) in the same reaction, in which a restriction enzyme cleavage site was inserted into two pairs of species-specific primers. This method permits to distinguish different pathogens simultaneously, owing to the subsequent restriction enzyme analysis. mLAMP has undergone numerous modifications and improvements, but which require the use of additional and more complex equipment. An example is represented by real time mLAMP. This approach permits the real-time detection of 1–4 target sequences in a single LAMP reaction tube, using a standard real-time fluorimeter, with standard LAMP primers that contain a quencher-fluorophore duplex region, which, upon strand separation, results in a gain of fluorescent signal [174]. Multiplex LAMP assays are now available for simultaneous detection of plant viruses [28,39].

### 9.2. Micro LAMP (μLAMP)

The LAMP assay can be integrated in a microfluidic chip either for read-out by the naked-eye (insoluble by-product pyrophosphate) or for measurement by an optic sensor; it requires a small sample volume (0.4 μL), with high sensitivity and specificity. It is possible to adapt the system to a real-time quantitative μLAMP by integration of optical fibers within the chip [175].

### 9.3. Digital LAMP (dLAMP)

In 2012, a digital LAMP method was developed using a sample self-digitization (SD) chip, demonstrating the first on-chip loop-mediated DNA amplification in a digital format [176]. This system allows to detect relative changes in template concentration, as well as absolute quantification of template numbers; the sample is directly pipetted into the chip, and a simple pump head and constant air pressure induce sample discretization for the subsequent isothermal amplification. dLAMP allows absolute and relative DNA quantification with good precision, within an incubation period of 70 min at 60–65 °C. In order to simplify and reduce effective costs, Lin and coworkers (2019) [177] reported a new dLAMP method that uses a commercial track-etched polycarbonate (PCTE) membrane instead of a chip, without the need for specialized equipment. This membrane system could be applied for point-of-care testing and to perform a flexible and simplified digital quantification.

### 9.4. LAMP Combined with Lateral Flow Assay (LFA)

**Figure 6:**
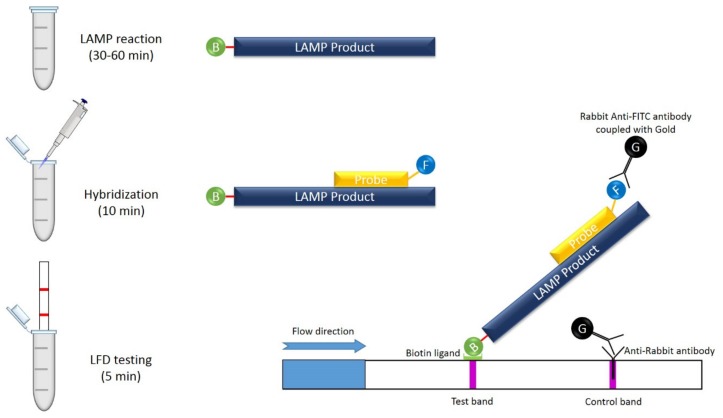
LAMP combined with lateral flow assay (LFA): the LAMP reaction is performed using a biotinylated forward inner primer (FIP) primer. After 30–60 min of initial incubation at constant temperature (60–65° C), a specific probe labelled with FITC (fluorescein isothiocyanate) is added to the reaction mixture and incubated for 10 min at the same temperature; in this step, dual-labeled LAMP product is produced. Subsequently, the reaction mixture is mixed with a detection buffer containing rabbit anti-FITC antibodies coupled with colloidal gold, and the LFD (Lateral-Flow Dipstick) strip is inserted into the tube. In a positive sample, the LAMP product complex moves through the LFA pad (an absorbent pad or strip) and binds with anti-FITC antibodies. The results can be read in a few minutes, just visualizing the pad. In the case of a negative reaction, no products are generated. An anti-rabbit antibody at the test control band retains some of the unbound gold conjugated antibody and produces a band that should always be visible.

This assay is similar in sensitivity to the conventional agarose gel electrophoresis, described above, but more rapid and easy to use, and does not require specialized equipment, such as a thermal cycler. This method is based on DNA hybridization technology and antigen–antibody reactions. In detail, a set of four primers is used: two outer primers (F3 and B3), as well as a biotin-labelled FIP and BIP. A the 5′ end, an FITC (fluorescein isothiocyanate)-labelled DNA probe is added, after LAMP reaction, for the lateral flow assay. The biotinylated LAMP product is hybridized with an FITC-labelled specific probe. The resulting DNA complex could be visualized as a purple band at the strip test line within 5 min of sample exposure. The LAMP product complex moves through the LFA pad (an absorbent pad or strip) and binds with anti-FITC antibodies (Figure 6). The results can be read in a few minutes, just visualizing the pad [178,179]. This system is well suited for in-field use, significantly reducing the time for diagnosis, as no sophisticated equipment is needed. Of all the systems described so far, this represents the most interesting portable system.

### 9.5. Immunocapture-RT-LAMP (IC-RT-LAMP)

To facilitate rapid on-site detection, Selvaraj and coworkers (2019) [38] improved an RT-LAMP assay for citrus tristeza virus (CTV) detection by initial capturing CTV virions from citrus crude leaf sap using CTV-IgG. This procedure allows to eliminate the RNA extraction step, reducing time and cost, and maintaining great sensibility and robustness. In fact, IC-RT-LAMP can be performed within an hour and a half directly in the field by unspecialized personnel.

### 9.6. Electric LAMP (eLAMP)

The eLAMP is an informatic device designed to improve the efficiency of testing putative LAMP primers on a target gene sequences using a Practical Extraction and Report Language (PERL) script with Tk graphical interface that electronically simulates the LAMP assay; it could be helpful to determine the possibility of using existing primer to amplify newly discovered sequence variants [180].

### 9.7. Lab-on-a-Disc (LoaD) LAMP Platform

This highly integrated “Lab-on-a-Disc” (LoaD) system consists of a fully integrated compact device that permits automated detection of plant pathogens by quantitative LAMP amplification. The platform consists of a disposable cartridge disc, which rotates on its axis to generate a centrifugal field; the resulting pressure head pumps the sample from the disc center to its periphery. In detail, samples are directly processed, purified, and subsequently mixed with LAMP reagents by digital pulse-actuated dissolvable-film valves. This custom instrument incorporates modules for heating and fluorescent detection. This all-in-one system shows a good potential for decentralized and on-site applications, such as cancer diagnostics and food quality monitoring. This system was developed for the detection of *Botrytis cinerea* [181], but can be easily adapted for the detection of viruses and viroids on plant material.

### 9.8. Lyophilized LAMP

In this LAMP version, all of the reagents are combined into a single mixture, called the lyophilized LAMP mix, in some cases associated with a closed amplification and detection system, in order to simplify and make the LAMP process more rapid; the user only needs to add water and sample or DNA/RNA template into the lyophilized mix before to carry out the incubation at the desired temperature. Many lyophilized LAMP kits are commercially available for rapid diagnosis of many diseases, making it usable under field conditions, facilitating the provision of the an-hour testing strategy in even the most remote rural health facilities, and achieving great diagnostic levels in resource-poor areas [182,183]. These kits are usually associated with portable thermal cyclers, which allow wireless connection with mobile devices (such as smartphones or tablets) for viewing data in real time. This system can also be used in the agricultural and phytosanitary field, especially if associated with decision support tools, in order to diagnose the possible presence of a plant pathogen in short times [184].

### 9.9. FRET-Based Assimilating Probe for Sequence-Specific Real-Time Monitoring of LAMP

This technique is based on the use of a fluorescence resonance energy transfer (FRET)-based probe. This technique permits the specific detection and target quantification of LAMP products from field samples [185]. In this LAMP version, for real-time sequence-specific monitoring, a pair of labeled oligonucleotide probes is used, collectively named the “assimilating probe”, following the principles of the FRET technique. The operating principle of the probe pair is analogous to a “molecular zipper”; a quenching strand is displaced from a partially complementary fluorescent strand during the DNA synthesis process. A molecular zipper is a fluorescent probe construct, which is used for quantifying target DNA by real-time monitoring ramification amplification (RAM) or rolling-circle amplification (RCA) reactions. This particular probe presents a very low background fluorescence owing to the strong interaction between two strands. When it is incorporated into the RAM products, its double strand region is opened by displacement, and then its fluorophore emits a fluorescent signal [186].

A great advantage of using this technology lies in reducing the potential error deriving from the visual detection of LAMP products. In addition, it improves the accuracy of pathogens detection, and allows their quantification at the same time. Several portable devices based on this technology are commercially available, for example, “Bioranger” (Diagenetix, Inc., Honolulu, HI, USA) and “Genie II and III” (Optigene Ltd., West Sussex, UK), which are used by growers or crop consultants, especially in nursey or phytosanitary program activities [164].

## 10. Advantages and Drawbacks of LAMP Assay

Among all the techniques based on the nucleic acid amplification, the LAMP assay, for its characteristics, high robustness, simplicity, and applicability in a resource-limited context, is widely used as an excellent diagnostic method that could also be used in developing countries where many plant diseases are endemic [22].

Other advantages of this technique include the following:

- Compared with other RNA/DNA amplification methods (i.e., PCR, RT-PCR, and RT-qPCR), LAMP shows a similar sensitivity and specificity [187].
- A typical LAMP test is rapid, and it is completed in about one hour; if loop primers are used, it requires no more than 30 min [24].
- LAMP works at a constant temperature, thanks to the high strand displacement activity of *Bst* polymerase [15].
- LAMP only needs a water bath or a block heater that can be used under field condition [15,156,188]. Furthermore, it does not require specialized personnel.
- When combined with reverse transcription, amplification of RNA sequences can be carried out in a LAMP assay with high efficiency.
- The robustness of the LAMP allows to also analyze unprocessed samples, which can be used as a template, as the activity of the *Bst* polymerase is not influenced by the presence of inhibiting substances. For example, in plant virus diagnosis, it could be possible to use direct crude plant extracts in order to avoid total RNA or DNA extraction, shortening the processing time, allowing the simultaneous analysis of multiple samples, and drastically reducing the total cost for single analysis [12].
- The result visualization can be performed with naked-eye or real-time methods, through the use of SYBR Green I, EtBr, HNB, or Calcein [171]. There is no need for post-amplification processing, reducing the time and the high risk of contamination.
- The detection limit of LAMP assay is comparable to an end point PCR, while this assay is higher compared with other isothermal amplification techniques such as NASBA, 3SR, and SDA, all of which having a limit of detection of less than 10 copies [15].

The major constraint of the LAMP assay is the proper designing of the primer. For this reason, multiplexing approaches for LAMP are less developed than for PCR. Furthermore, as the products of LAMP are a mixture of stem–loop DNAs and a cauliflower-like structure, the technique is not suitable for cloning purposes. The excellent sensitivity of the LAMP method makes it vulnerable to contamination, in fact, one of the principals limiting factors of the LAMP technique is represented by the possible contaminations that occur when electrophoresis is carried out for the visualization of the results. In fact, the LAMP product is so robust that it does not easily degrade, and this implicates the possibility of carrying over the contamination [189]. This problem can be easily overcome by pre-incubating the LAMP mix with Uracil-N-glycosylase (UNG) [190]. As described above, another very easy method to prevent carry-over contamination is to use DNA binding dyes or metal ion indicators in the LAMP mixture. With this procedure, it is not necessary to open the tube when the amplification reaction has been completed and the detection is performed as a single “closed-tube” technology [191]. Finally, to control contaminations, master mix preparation should be made on ice in less than 30 min [33].

LAMP is an effective tool for diagnosis and prevention for emergence of new and re-emergence of existing diseases/pathogens. For this reason, in the last 20 years, it has spread considerably throughout the world and been successfully applied in molecular detection and diagnostics, especially in developing countries, for the detection of human and plant pathogens, genetically modified organisms (GMO) [192], embryo sex identification [193], and cancer detection [194].

In plant pathology, it is necessary to avoid the spread of different pathogens and the introduction of new pathogens or pests in a new area, in order to carry out new rapid molecular methods [7]. Up to now, many reports about the LAMP method for detecting different plant pathogens are available. As reported previously, many LAMP-based methods were developed for different plant viral diseases, important plant bacterial diseases, phytoplasma, and fungi.

In summary, the LAMP technique represents an important methodology to avoid the spread of endemic diseases or the introduction of dangerous pathogens [4,5,195,196,197,198] into new geographic areas.

## Figures and Tables

**Table 1:** Loop-mediated isothermal amplification (LAMP) protocols developed for different plant viruses and viroids.

Species	Acronym	Genome	Family	Genus	Reference
*Abaca bunchy top virus*	ABTV	ssDNA(+)	*Nanoviridae*	*Babuvirus*	[49]
*Apple chlorotic leaf spot virus*	ACLSV	ssRNA(+)	*Betaflexiviridae*	*Trichovirus*	[50,51]
*Apple scar skin viroid*	ASSVd	circRNA	*Pospiviroidae*	*Apscaviroid*	[52]
*Apple stem pitting virus*	ASPV	ssRNA(+)	*Betaflexiviridae*	*Foveavirus*	[51]
*Arabis mosaic virus*	ArMV	ssRNA(+)	*Secoviridae*	*Nepovirus*	[53]
*Banana bract mosaic virus*	BBMV	ssRNA(+)	*Potyviridae*	*Potyvirus*	[54]
*Banana bunchy top virus*	BBTV	ssDNA(+)	*Nanoviridae*	*Babuvirus*	[49,55]
*Banana streak virus*	BSV	dsDNA-RT	*Caulimoviridae*	*Badnavirus*	[56]
*Barley stripe mosaic virus*	BSMV	ssRNA(+)	*Virgaviridae*	*Hordeivirus*	[57]
*Barley yellow dwarf virus*	BYDV	ssRNA(+)	*Luteoviridae*	*Luteovirus*	[58]
*Bean common mosaic necrosis virus*	BCMNV	ssRNA(+)	*Potyviridae*	*Potyvirus*	[59]
*Bean pod mottle virus*	BPMV	ssRNA(+)	*Secoviridae*	*Comovirus*	[60]
*Beet curly top virus*	BCTV	Circ-ssDNA(+/−)	*Geminiviridae*	*Curtovirus*	[61]
*Beet mild curly top virus*	BMCTP	Circ-ssDNA(+/−)	*Geminiviridae*	*Curtovirus*	[61]
*Beet necrotic yellow vein virus*	BNYVV	ssRNA(+)	*Benyviridae*	*Benyvirus*	[62]
*Beet severe curly top virus*	BSVTV	Circ-ssDNA(+/−)	*Geminiviridae*	*Curtovirus*	[61]
*Cassava brown streak virus*	CBSV	ssRNA(+)	*Potyviridae*	*Ipomovirus*	[63]
*Chilli veinal mottle virus*	ChiVMV	ssRNA(+)	*Potyviridae*	*Potyvirus*	[64]
*Chinese wheat mosaic virus*	CWMV	ssRNA(+)	*Virgaviridae*	*Furovirus*	[65]
*Chrysanthemum chlorotic mottle viroid*	CCMVd	circRNA	*Avsunviroidae*	*Pelamoviroid*	[66]
*Chrysanthemum stem necrosis virus*	CSNV	ssRNA(+/−)	*Tospoviridae*	*Orthotospovirus*	[67]
*Chrysanthemum stunt viroid*	CSVd	CircRNA	*Pospiviroidae*	*Pospiviroid*	[68]
*Chrysanthemum virus B*	CVB	ssRNA(+)	*Betaflexiviridae*	*Carlavirus*	[68]
*Citrus leaf blotch virus*	CLBV	ssRNA(+)	*Betaflexiviridae*	*Citrivirus*	[69]
*Citrus tristeza virus*	CTV	ssRNA(+)	*Closteroviridae*	*Closterosvirus*	[38,70,71]
*Citrus yellow mosaic virus*	CYMV	dsDNA-RT	*Caulimoviridae*	*Badnavirus*	[72]
*Coconut cadang-cadang viroid*	CCCVd	CircRNA	*Pospiviroidae*	*Cocadviroid*	[73]
*Columnea latent viroid*	CLVd	CircRNA	*Pospiviroidae*	*Pospiviroid*	[74]
*Cucumber green mottle mosaic virus*	CGMV	ssRNA(+)	*Virgaviridae*	*Tobamovirus*	[75]
*Cucumber mosaic virus*	CMV	ssRNA(+)	*Bromoviridae*	*Cucumovirus*	[76,77,78]
*Cucurbit chlorotic yellows virus*	CCYV	ssRNA(+)	*Closteroviridae*	*Crinivirus*	[79]
*Cucurbit leaf crumple virus*	CuLCrV	Circ-ssDNA(+/−)	*Geminiviridae*	*Begomovirus*	[80]
*Cymbidium mosaic virus*	CymMV	ssRNA(+)	*Alphaflexiviridae*	*Potexvirus*	[81]
*Fig mosaic virus*	FMV	ssRNA(−)	*Fimoviridae*	*Emaravirus*	[82]
*Grapevine leafroll-associated virus 3*	GLRaV-3	ssRNA(+)	*Closteroviridae*	*Ampelovirus*	[27]
*Grapevine red blotch virus*	GRBD	ssDNA(+/−)	*Geminiviridae*	*Grablovirus*	[37]
*Impatiens necrotic spot virus*	INSV	ssRNA	*Bunyaviridae*	*Tospovirus*	[83]
*Japanese soil-borne wheat mosaic virus*	JSBWMV	ssRNA(+)	*Virgaviridae*	*Furovirus*	[65]
*Japanese yam mosaic virus*	JYMV	ssRNA(+)	*Potyviridae*	*Potyvirus*	[84]
*Lettuce necrotic yellows virus*	LNYV	ssRNA(−)	*Rhabdoviridae*	*Cytorhabdovirus*	[85]
*Lily mottle virus*	LMoV	ssRNA(+)	*Potyviridae*	*Potyvirus*	[86]
*Lily symptomless virus*	LSLV	ssRNA(+)	*Betaflexiviridae*	*Carlavirus*	[87,88]
*Little cherry virus 1*	LChV-1	ssRNA(+)	*Closteroviridae*	*Velarivirus*	[89]
*Maize chlorotic dwarf virus*	MCDV	ssRNA(+)	*Sequiviridae*	*Waikavirus*	[90]
*Maize chlorotic mottle virus*	MCMV	ssRNA(+)	*Tombusviridae*	*Machlomovirus*	[91]
*Melon necrotic spot virus*	MNSV	ssRNA(+)	*Tombusviridae*	*Carmovirus*	[92]
*Melon yellow spot virus*	MYSV	ssRNA(+/−)	*Bunyaviridae*	*Tospovirus*	[93]
*Mesta yellow vein mosaic virus*	MeYVMV	Circ-ssDNA(+/−)	*Geminiviridae*	*Begomovirus*	[94]
*Milk vetch dwarf virus*	MDV	ssDNA(+)	*Nanoviridae*	*Nanovirus*	[95]
*Mirafiori lettuce big-vein virus*	MiLBVV	ssRNA(−)	*Ophioviridae*	*Ophiovirus*	[96]
*Onion yellow dwarf virus*	OYSV	ssRNA(+)	*Potyviridae*	*Potyvirus*	[97]
*Papaya leaf distortion mosaic virus*	PLDMV	ssRNA(+)	*Potyviridae*	*Potyvirus*	[98]
*Papaya ringspot virus*	PRSV	ssRNA(+)	*Potyviridae*	*Potyvirus*	[99]
*Peach latent mosaic viroid*	PLMVd	CircRNA	*Avsunviroidae*	*Pelamoviroid*	[100]
*Pepino mosaic virus*	PepMV	ssRNA(+)	*Alphaflexiviridae*	*Potexvirus*	[101,102]
*Pepper chat fruit viroid*	PCFVd	CircRNA	*Pospiviroidae*	*Pospiviroid*	[103]
*Pepper mottle virus*	PepMoV	ssRNA(+)	*Potyviridae*	*Potyvirus*	[104]
*Pepper yellow leaf curl Indonesia virus*	PepYLCIDV	Circ-ssDNA(+/−)	*Geminiviridae*	*Begomovirus*	[39]
*Piper yellow mottle virus*	PYMoV	dsDNA	*Caulimoviridae*	*Badnavirus*	[76]
*Plantago asiatica mosaic virus*	PlAMV	ssRNA(+)	*Alphaflexiviridae*	*Potexvirus*	[105]
*Plum pox virus*	PPV	ssRNA(+)	*Potyviridae*	*Potyvirus*	[30]
*Potato leafroll virus*	PLRV	ssRNA(+)	*Luteoviridae*	*Polerovirus*	[106,107]
*Potato spindle tuber viroid*	PSTVd	Circ-ssRNA	*Pospiviroidae*	*Pospiviroid*	[108]
*Potato virus X*	PVX	ssRNA(+)	*Alphaflexiviridae*	*Potexvirus*	[109,110]
*Potato virus Y*	PVY	ssRNA(+)	*Potyviridae*	*Potyvirus*	[111,112]
*Prunus necrotic ringspot virus*	PNRSV	ssRNA(+)	*Bromoviridae*	*Ilarvirus*	[113]
*Rice black-streaked dwarf virus*	RBSDV	dsRNA	*Reoviridae*	*Fijivirus*	[114,115]
*Rice dwarf virus*	RDV	dsRNA	*Reoviridae*	*Phytoreovirus*	[114,116]
*Rice gall dwarf virus*	RGDV	dsRNA	*Reoviridae*	*Phytoreovirus*	[114,116]
*Rice grassy stunt virus*	RGSV	ssRNA(−)	*Phenuiviridae*	*Tenuivirus*	[114,116]
*Rice ragged stunt virus*	RRSV	dsRNA	*Reoviridae*	*Oryzavirus*	[114,115,116,117]
*Rice stripe virus*	RSV	ssRNA(−)	*Phenuiviridae*	*Tenuivirus*	[114,116]
*Rice transitory yellowing virus*	RTYV	ssRNA(−)	*Rhabdoviridae*	*Nucleorhabdovirus*	[114,116]
*Rice tungro bacilliform virus*	RTBV	dsDNA-RT	*Caulimoviridae*	*Tungrovirus*	[114,116]
*Rice tungro spherical virus*	RTSV	ssRNA(+)	*Secoviridae*	*Waikavirus*	[114,116]
*Sorghum mosaic virus*	SrMV	ssRNA(+)	*Potyviridae*	*Potyvirus*	[118]
*Southern rice black-streaked dwarf virus*	SRBSDV	dsRNA	*Reoviridae*	*Fijivirus*	[119]
*Southern tomato virus*	STV	dsRNA	*Amalgaviridae*	*Amalgavirus*	[35]
*Squash leaf curl virus*	SLCV	Circ-ssDNA(+/−)	*Geminiviridae*	*Begomovirus*	[120]
*Squash mosaic virus*	SqMV	ssRNA(+)	*Comoviridae*	*Comovirus*	[121]
*Strawberry latent ringspot virus*	SLRSV	ssRNA(+)	*Secoviridae*	Unassigned	[122]
*Sugarcane mosaic virus*	SCMV	ssRNA(+)	*Potyviridae*	*Potyvirus*	[118,123]
*Sugarcane streak mosaic virus*	SCSMV	ssRNA(+)	*Potyviridae*	*Poacevirus*	[123]
*Sugarcane yellow leaf virus*	SCYLV	ssRNA(+)	*Luteoviridae*	*Polerovirus*	[124]
*Tobacco etch virus*	TEV	ssRNA(+)	*Potyviridae*	*Potyvirus*	[125]
*Tobacco mosaic virus*	TMV	ssRNA(+)	*Virgaviridae*	*Tobamovirus*	[125,126]
*Tobacco streak virus*	TSV	ssRNA(+)	*Bromoviridae*	*Ilarvirus*	[127]
*Tobacco vein banding mosaic virus*	TVBMV	ssRNA(+)	*Potyviridae*	*Potyvirus*	[125]
*Tomato aspermy virus*	TAV	ssRNA(+)	*Bromoviridae*	*Cucumovirus*	[128]
*Tomato black ring virus*	TBRV	ssRNA(+)	*Secoviridae*	*Nepovirus*	[129]
*Tomato brown rugose fruit virus*	ToBRFV	ssRNA(+)	*Virgaviridae*	*Tobamovirus*	[130]
*Tomato chlorosis virus*	ToCV	ssRNA(+)	*Closteroviridae*	*Crinivirus*	[131,132]
*Tomato chlorotic spot virus*	TCSV	ssRNA(+/−)	*Tospoviridae*	*Orthotospovirus*	[133]
*Tomato leaf curl Bengaluru virus*	ToLCBaV	Circ-ssDNA(+/−)	*Geminiviridae*	*Begomovirus*	[134]
*Tomato leaf curl New Delhi virus*	ToLCNDV	Circ-ssDNA(+/−)	*Geminiviridae*	*Begomovirus*	[39,135,136]
*Tomato necrotic stunt virus*	ToNSV	ssRNA(+)	*Potyviridae*	*Potyvirus*	[137]
*Tomato spotted wilt virus*	TSWV	ssRNA(+/−)	*Tospoviridae*	*Orthotospovirus*	[138]
*Tomato torrado virus*	ToTV	ssRNA(+)	*Secoviridae*	*Torradovirus*	[32]
*Tomato yellow leaf curl Kanchanaburi virus*	TYLCKaV	Circ-ssDNA(+/−)	*Geminiviridae*	*Begomovirus*	[39]
*Tomato yellow leaf curl virus*	TYLCV	Circ-ssDNA(+/−)	*Geminiviridae*	*Begomovirus*	[139]
*Turnip mosaic virus*	TuMV	ssRNA(+)	*Potyviridae*	*Potyvirus*	[140]
*Turnip yellows virus*	TuYV	ssRNA(+)	*Luteoviridae*	*Polerovirus*	[141]
*Ugandan cassava brown streak virus*	UCBSV	ssRNA(+)	*Potyviridae*	*Ipomovirus*	[63]
*Watermelon mosaic virus*	WMV	ssRNA(+)	*Potyviridae*	*Potyvirus*	[121]
*Wheat streak mosaic virus*	WSMV	ssRNA(+)	*Potyviridae*	*Tritimovirus*	[142]
*Wheat yellow mosaic virus*	WYMV	ssRNA(+)	*Potyviridae*	*Bymovirus*	[65,143]
*Yam mosaic virus*	YMV	ssRNA(+)	*Potyviridae*	*Potyvirus*	[144]
*Zucchini yellow mosaic virus*	ZYMV	ssRNA(+)	*Potyviridae*	*Potyvirus*	[121,145]
